# HCV Virus and Lymphoid Neoplasms

**DOI:** 10.1155/2011/717951

**Published:** 2011-07-05

**Authors:** Yutaka Tsutsumi, Shinichi Ito, Reiki Ogasawara, Kazuhiro Kudo, Junji Tanaka, Masahiro Asaka, Masahiro Imamura

**Affiliations:** ^1^Department of Hematology, Hakodate Municipal Hospital, 1–10-1 Minato-cho, Hakodate 041-8680, Japan; ^2^Department of Clinical Pathology, Hakodate Municipal Hospital, 1–10-1 Minato-cho, Hakodate 041-8680, Japan; ^3^Department of Hematology and Oncology, Hokkaido University Graduate School of Medicine, Sapporo 60-8638, Japan; ^4^Department of Gastroenterology, Hokkaido University Graduate School of Medicine, Sapporo 60-8638, Japan

## Abstract

Hepatitis C virus (HCV) is one of the viruses known to cause hepatic cancer. HCV is also believed to be involved in malignant lymphoma. In this paper, we investigated characteristics of malignant lymphoma cases that were anti-HCV antibody (HCV-Ab) positive. We were able to perform pathological examinations on 13 out of 14 HCV-positive cases. Of these, lymphoid tissues of 10 stained positive for HCV-Ab. There was no significant correlation between the degree of HCV staining and the rate of recurrence or resistance to treatment. However, there did appear to be a consistent decrease in the amount of HCV-RNA between pre- and posttreatment among HCV-Ab-positive cases; that is, treatment-resistant cases that exhibited resistance from the first treatment and recurrent cases more frequently had a higher HCV level at treatment termination compared to the pretreatment level. This suggests that the HCV virus either accelerates oncogenesis by direct interaction with B cells or indirectly affects lymphoma prognosis.

## 1. Introduction

Since the discovery of hepatitis C virus (HCV) in 1989, the causal relationship between HCV and hepatic cancer has been proven through various case control studies, and it has been suggested that the risk of hepatic cancer in the HCV-infected population is 23–35-times higher than in uninfected healthy individuals [1, 2]. In some cases, however, HCV can cause hepatic carcinoma without leading to the development of hepatic cirrhosis. The mechanism by which HCV causes cancer is therefore not clear [2]. Several reports indicate a correlation between malignant lymphoma and HCV infection [3, 4] whereas other reports showed no correlation [1]. A definitive answer on the relationship between HCV infection and malignant lymphoma is therefore lacking. We previously reported recurrence of malignant lymphoma in clinical cases which had altered anti-HCV antibody (HCV-Ab) levels or in those with abnormally high HCV-RNA level during chemotherapy treatment for lymphoma [5, 6]. In this study, we analyzed the incidence of malignant lymphoma among HCV-Ab-positive cases and characteristics and prognoses observed in our hospital, and we compared the results with previously reported cases.

## 2. Patients and Methods

### 2.1. Patients

Analysis of HCV-Ab-positive patients was conducted between October 2002 and December 2010, during the period that rituximab was marketed and available in Japan. One hundred eighty cases of B lymphocyte non-Hodgkin's lymphoma (NHL) were used as a control group and were treated around the same period of time. The HCV-Ab positive group consisted of 14 patients between 43 and 90 years old with a median age of 72 years. These cases included 12 with diffuse large B cell lymphoma, 1 with mantle cell lymphoma, and 1 with EB virus-related lymphoproliferative disorder. Eight cases were of grades III and IV, and no gender differences were observed. Eight cases (57%) were either treatment resistant at the time of first treatment or were recurrent cases. The median age of the control group was 66 years and the male/female ratio was approximately the same. However, the incidence of diffuse large B cell lymphoma in this group was 60% and was seen to be less than the anti-HCV-Ab positive group. There were seventy-one cases (31%) with first treatment resistance or recurrence in the control group, and the ratio was less than that seen in the anti-HCV-Ab positive group (Table 1).

### 2.2. HCV Immunohistochemical Staining of Lymphoid Tissues

 Specimens consisted of lymph nodes (7 cases), stomach (2), tonsil (1), rectum (1), soft tissue (1), and orbit (1). Specimens were fixed in 10% neutral-buffered formalin, embedded in paraffin. Diagnosis for malignant lymphoma was made by HE and immunohistochemical staining. 

**Figure 1:**
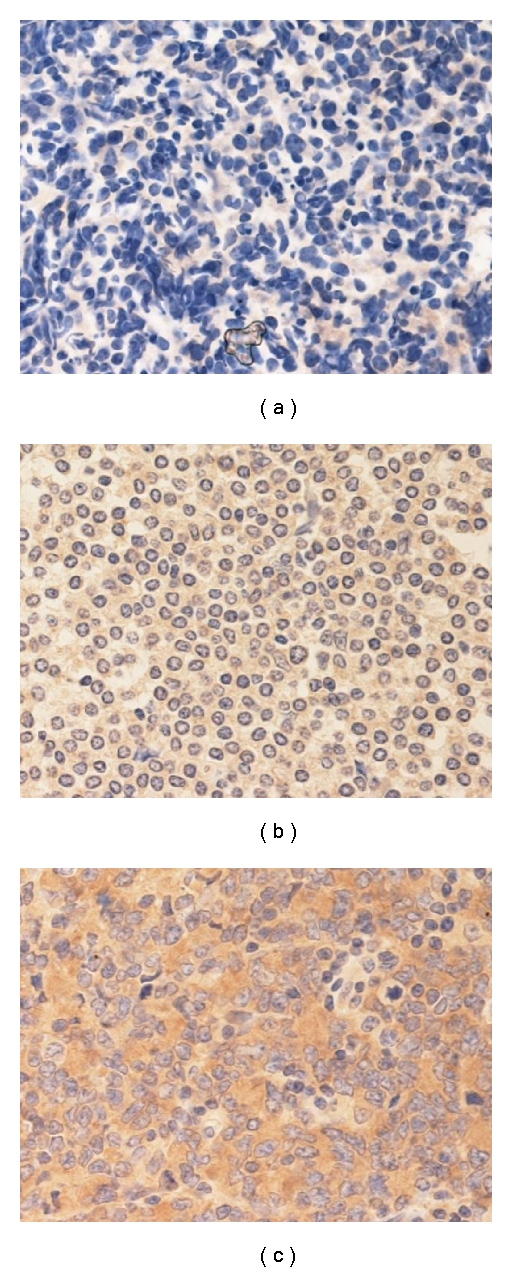
Immunohistochemical stains of lymphoma tissues (a) negative, (b) weakly positive, and (c) strongly positive.

 To evaluate HCV positivity of lymphoma cells, immunohistochemical staining was performed using monoclonal antibodies to hepatitis C virus (NS3) NCL-HCV-NS3 (Leica Microsystems, Weltzlar, Germany). HCV-specific reactions were detected with commercially available Histofine simple stain (Nichirei Co., Tokyo, Japan). Immunoreactivity was graded as negative, weakly positive, and strongly positive (Figures 1(a), 1(b), and 1(c)).

### 2.3. Survival

Survival and progression-free survival (PFS) probabilities were estimated with the Kaplan-Meier Method. Overall survival (OS) was calculated from the date of diagnosis to the date of a patient's death for any reason or to last followup. PFS and relapse were determined with cumulative incidence estimates, treating relapse and death as competing risk events. All time-to-event endpoints were censored at the time of last contact. 

## 3. Results

### 3.1. Pathological Examination by Immunohistochemical Staining with Anti-HCV Antibody

Immunohistopathological analysis of anti-HCV-Ab positive lymphoma cells revealed 5 strongly positive, 5 weakly positive, and 3 negative cases. These observations indicate that the growth and development of lymphoma cells may be associated with the presence of HCV. The level of HCV staining was also analyzed to determine if a correlation to treatment resistance or recurrence potential existed. Although the number of cases was too limited to obtain a clear conclusion, 3 out of 5 strongly positive cases were also treatment resistant or recurrent cases. Among 5 weakly positive cases, 3 were treatment resistant or recurrent cases. There were only 3 negative cases, 2 of which were treatment-resistant or recurrent cases. It would therefore be difficult to predict treatment-resistance or recurrence based on immunoreactivity. These results indicate that HCV directly interacts with B lymphocytes and causes lymphoma, however, the outcome cannot be predicted.

### 3.2. Characteristics of Anti-HCV-Ab Positive Cases

**Figure 2:**
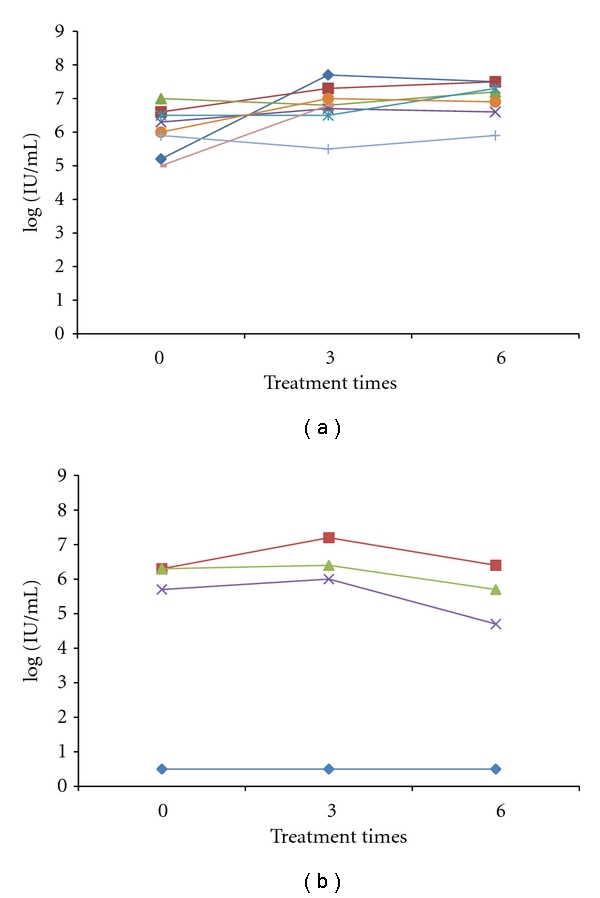
(a) The change in HCV-RNA in cases with treatment resistance or recurrence. (b) The change in HCV-RNA in cases that maintained remission. In many cases that showed treatment resistance or recurrence, HCV-RNA levels transiently increased after the third anti-cancer treatment compared to pretreatment levels. Although in two cases HCV-RNA level decreased compared to pre-treatment level, values were higher than the pre-treatment level after the sixth treatment. In contrast, in some of the cases that maintained remission, HCV-RNA levels increased transiently after the third treatment, but after the sixth treatment decreased to lower than pre-treatment levels.

One patient with grade I disease who had undergone surgery was excluded from the study and the remaining 13 cases were analyzed. Among these, 11 had liver disorder and 2 exceeded Grade III. Increased bilirubin was also seen in 2 cases. HCV-RNA level increased in 10 cases during the treatment period but only 9 of them appeared to cause hepatic toxicity. In 5 cases, HCV-RNA transiently decreased after hepatic toxicity but never become undetectable. The level of HCV-RNA prior to or just before treatment was compared with that on the last day of visit (including the treatment period). A decrease in HCV-RNA was considered to have occurred when the ratio of HCV-RNA on the last visit/pre-treatment HCV-RNA was less than 1.0.  Figure 2 shows the changes in HCV-RNA from these cases. HCV was measured pre-, postthird, and postsixth treatments. Many cases showed an increase in HCV-RNA afterthe third treatment. In cases with treatment resistance or recurrence, HCV-RNA afterthe sixth treatment was never lower than the pre-treatment level. In contrast, although HCV-RNA increased after the third treatment in many cases which maintained complete remission, most cases among these showed a decrease in HCV-RNA after the sixth treatment. In cases with constantly low levels of HCV-RNA, lymphoma tissues showed positive staining for anti-HCV antibody, however, HCV-RNA was not detectable throughout the treatment course. In one case that maintained complete remission, interferon treatment was added following anticancer treatment and HCV-RNA became nondetectable. The analysis showed that there was no treatment resistance or recurrence among cases with decreased HCV-RNA whereas treatment resistance or recurrence did occur in cases with increased HCV-RNA (Table 2).

### 3.3. Overall Survival and Progression Free Survival

**Figure 3:**
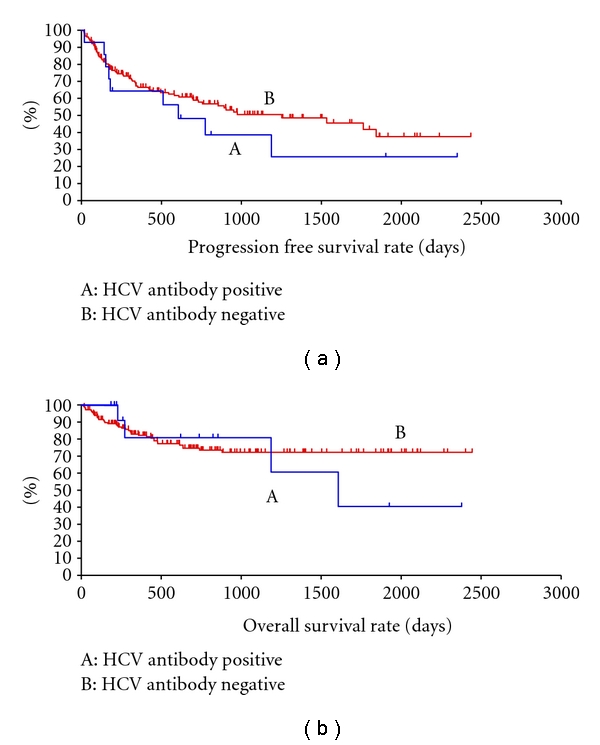
Progression free survival (a) and overall survival (b) curves of non-Hodgkin's lymphoma patients who were anti-HCV-antibody negative (B) and anti-HCV antibody positive (A). Both survival parameters were lower in anti-HCV antibody positive patients, however, no significant difference was seen when comparing these two groups.

Figure 3 shows progression free survival and overall survival in our institute calculated by the Kaplan-Meier method. There seemed to be no significant difference in progression free survival between the anti-HCV-Ab-positive group and the control group, however, overall survival appears to be higher in anti-HCV-Ab-negative group. Due to the small sample size of anti-HCV-Ab-positive cases, however, the difference did not reach statistical significance.

## 4. Discussion

Various reports have suggested an association between HCV and malignant tumors, particularly hepatic cancer. A correlation between HCV and B cell lymphoma was also reported recently [7–12]. We also observed recurrent cases of B cell lymphoma which were associated with a rapid increase in HCV viral load, indicating a role for HCV in B cell lymphoma progression [5]. In this study, we investigated the correlation between anti-HCV-Ab positivity and B cell lymphoma by performing immunohistochemical staining on pathological samples. Almost all cases showing weak or strongly positive staining were also HCV positive. Although lymphoid cells may simply be positive for HCV antigen, this result also may indicate a possible correlation between HCV and lymphoma. Although the majority of cases in this study were diffuse large B cell lymphoma, an association between HCV and marginal zone lymphoma, small lymphocytic lymphoma/chronic lymphocytic lymphoma, lymphoplasmacytic lymphoma and follicular center lymphoma has also been reported [13–15]. Several studies also indicate a correlation between HCV and B cells. For example, it has been reported that HCV-RNA can be maintained over a prolonged period in B cells and that activation-induced cytidine deaminase increases in these cells, which may affect lymphocyte growth [13, 16]. It is possible that the growth of B cells that utilize these particular signals may contribute to the progression of B cell lymphoma at the level of DNA replication, however, further studies are needed to clarify this relationship. There was, however, no correlation between the level of HCV staining and prognosis. Although it is possible that HCV is directly involved in lymphoma development, there seems to be no correlation between HCV infection of lymphoma cells and prognosis, and only indirect involvement of HCV is therefore suggested.

It was not possible to perform statistical analyses in this study due to the small number of anti HCV-Ab positive cases. Nevertheless, the overall survival data suggested a poorer prognosis in the anti HCV-Ab positive group compared to the HCV-Ab negative B cell lymphoma group. Previous reports have been inconsistent, however. One study reported no change in prognosis, including recurrence rate. Other studies reported a reduction in progression free survival, but no effect on overall survival. In additional studies, a reduction in both progression free survival and overall survival was reported [17–19]. In order to clarify these inconsistent observations, it is desirable to conduct experiments on patient groups with defined disease and analyzed under identical conditions.

We also compared the characteristics of B cell lymphoma patients to anti-HCV-Ab positivity with regard to the level of HCV-RNA. An increase in HCV-RNA was observed in 10 anti-HCV-Ab positive cases. Among these, 8 cases had a higher level of HCV-RNA at the completion of treatment compared to the level at initial visit (pre-treatment HCV-RNA level). As reported by Ennishi et al., although HCV viral load increased upon treatment of the lymphoma, in a majority of cases, the amount of virus decreased over 1–6 months following treatment. This indicates that, although few in number, cases occurred in which the amount of virus did not decrease to the pre-treatment level [17]. We also observed no reduction in HCV-RNA in some recurrent or treatment-resistant cases, when compared to the level at initial visit. There was also no recurrence seen in those in whom the level of HCV-RNA had declined at the completion of treatment compared to the first visit. This indicates that unless HCV-RNA returns to the pre-treatment level at the end of treatment, repeated recurrence can occur in some cases, indicating that it may be possible to predict prognosis based on the viral load at the end of treatment. In fact, it has been reported that ribavirin was effective in HCV-positive cases [16, 20, 21] and that there was a reduction in the development of lymphoma when interferon was effectively used for treatment [22]. Interferon has been shown to be effective for suppression of HCV and prevention of hepatic carcinoma, and it may also therefore be effective for malignant lymphoma. Decisions on treatment options should therefore include, for example, concurrent use of interferon from initial treatment and during maintenance therapy, as such treatments have the potential to prevent hepatic carcinoma as well. Although there are problems with such therapies, including a myelosuppressive effect due to concomitant drug use and high cost, the potential benefits warrant conduction of a large-scale clinical study.

Hepatic toxicity due to hepatitis B virus reactivation during rituximab treatment has been well recognized [23], and the possibility of similar severe hepatic toxicity resulting from treatment with rituximab in HCV-positive cases has also been discussed, although this is an unlikely event with normal chemotherapy [17]. We have also reported previously that anti-HCV-Ab declines while HCV-RNA increases during rituximab treatment, and that the level of HCV-RNA decreases when the interval of rituximab is extended [6]. We are therefore interested in large-scale studies conducted by other groups. In a large-scale clinical study, Ennishi et al., reported that hepatic toxicity was often found in HCV positive cases, which led to modification in treatment schedule [17]. Pitini et al., analyzed a small number of hepatitis cases arising from HCV reactivation. Although only 4 cases were analyzed in this study, HCV genotype II tended to be associated with hepatitis [24]. In our study, 11 out of 14 cases developed hepatitis, with one dying of hepatic failure. In this particular case, hepatic failure rapidly progressed during treatment. Since this patient also developed EBV infection, the patient may have had an immune-deficient background. Two cases of Grade III and higher hepatic toxicity were also observed. The frequency was not low, it is possible that hepatic toxicity was caused by an increased HCV level, as seen with HBV infection, and by removal of immune suppression. A decrease in HCV-RNA level after hepatic toxicity was observed in 5 cases in the present study and in 3 of these, the amount of HCV-RNA decreased to below pre-treatment levels. In these cases, no recurrence was observed. It may be possible to lower the recurrence rate by reducing HCV-RNA level if there is no delay in treatment. On the other hand, if hepatic toxicity occurs, the treatment interval may have to be extended and as a result, treatment efficacy may be reduced. Therefore, hepatic toxicity should be avoided after all. The use of rituximab may be effective if the risk level can be predicted via genotypic analysis of HCV and identification of other risk factors for hepatic toxicity. Although we were unable to analyze these parameters in the present study, a further largescale investigation is desirable. 

In conclusion, although a firm relationship between HCV and B cell lymphoma remains to be proven, there appears to be a definite correlation between the two. Since survival of anti-HCV-Ab positive lymphoma patients is not necessarily higher than overall survival in anti-HCV-Ab negative cases (it can in fact be lower), it may be possible to improve survival through the use of interferon treatment. The frequency is not low, and the use of rituximab may cause further severe hepatic toxicity. Recurrence and other problems may decrease if HCV-RNA level consistently declines and chemotherapy is applied as scheduled. However, this approach is not practical and severe hepatic toxicity caused by HCV reactivation should be avoided. In order to prevent HCV reactivation leading to hepatic toxicity, methods to identify risk factors which can be easily used at clinical sites are needed. Various questions remain to be answered such as (1) whether concomitant ribavirin or interferon should be used for current therapy, and if so, (2) whether they should be used from the beginning of treatment or at the end of existing treatment. There are no reports on chemotherapy with concurrent use of rituximab. Future clinical studies are needed to clarify these questions.

## Figures and Tables

**Table 1:** Characteristics of the patients.

	Anti-HCV-antibody positive	Anti-HCV antibody negative
Median age (range)	72 (43–90)	66 (19–99)
Sex (M/F)	7/7	99/81
Disease		
DLB	12	108
FL		43
MALT	1	11
MCL		11
BL		4
EB associated LPD	1	3
Stage	6	46
I-II		
III-IV	8	134
International prognostic index		
(1/L1/1H/H)	4/2/4/4	48/42/41/49
Recurrence/refractory	8	71

Abbreviations: HCV as hepatitis C virus; DLB as diffuse large B cell lymphoma; FL as follicular lymphoma; MALT as maltoma; MCL as mantle cell lymphoma; Burkitt lymphoma; LPD as Iymphoproliferative disease; L as low risk; LI as low-intermediate risk; IH as intermediate-high risk: H as high risk of international prognostic index.

**Table 2:** Tendency of the anti-HCV-antibody-positive patients.

Complication of hepatitis 11
AST/ALT
Grade I/II 9
above Grade III 2
T-Bil
Grade I/II 1
above Grade III 1
HCV RNA elevation during chemotherapy 10
HCV RNA elevation with hepatitis 9
Reduction of transient HCV RNA after hepatic toxicity 5
Recurrence or refractory
Last time/first time HCV RNA below 1.0 0/5
Last time/first time HCV RNA above 1.0 8/8

Abberiviatins: HCV as hepatitis C virus; T-Bil as total bilirubin; AST as aspartate aminotransferase; ALT as alanine transaminase.
